# Community plant height modulated by aridity promotes spatial vegetation patterns in Alxa plateau in Northwest China

**DOI:** 10.1002/ece3.9823

**Published:** 2023-02-14

**Authors:** Tian‐liang Cheng, Yan‐xia Pan, Xin‐ping Wang, Yan Li

**Affiliations:** ^1^ State Key Laboratory of Subtropical Silviculture Zhejiang A&F University Hangzhou China; ^2^ Northwest Institute of Eco‐Environment and Resources Chinese Academy of Sciences Lanzhou China

**Keywords:** aridity, community plant height, functional traits, patch size distribution, plant–plant interactions

## Abstract

Spatial vegetation patterns are associated with ecosystem stability and multifunctionality in drylands. Changes in patch size distributions (PSDs) are generally driven by both environmental and biological factors. However, the relationships between these factors in driving PSDs are not fully understood. We investigated 80 vegetation plots along an aridity gradient in the Alxa plateau, Northwest China. The sizes of vegetation patches were obtained from aerial images, and the heights of patch‐forming species were measured in the field. Soil samples were collected on the bare ground between patches for determination of physiochemical properties. Point pattern analysis was used to infer plant–plant interactions. A model selection procedure was employed to select the best predictors for the shape of PSDs and biological factors (vegetation total cover, community plant height, and plant–plant interactions). We then used structural equation modeling to evaluate the direct and indirect effects of environmental and biological factors on the shape of PSDs. In our study area, two types of PSDs coexisted, namely those that best fit to power law distributions and those that best fit to lognormal distributions. Aridity was the main environmental factor, while community mean height and competition between plants were the main biological factors for the shape of PSDs. As aridity and community mean height increased, power law‐like PSDs were exhibited, whereas competition led to deviations of PSDs from power laws. Aridity affected the shape of PSDs indirectly through changes in community mean height. Community mean height was correlated with competition, thereby indirectly affecting the shape of PSDs. Our results suggest the use of community functional traits as a link between the environment and plant–plant interactions, which may improve the understanding of the underlying mechanisms of PSD dynamics.

## Graphical abstract

Our work strengthens the relationship between community functional traits and spatial vegetation pattern. Our results suggest the use of community functional traits as a link between the environment and plant–plant interactions, which may improve the understanding of underlying mechanisms.

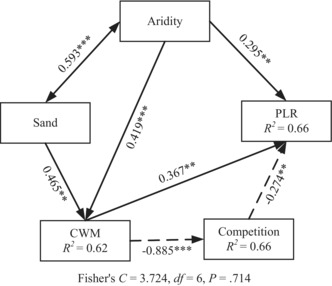

## 1 INTRODUCTION

Drylands typically exhibit spatial patterns mosaicked by distinct patches of bare soil and vegetation (Aguiar & Sala, 1999). In between these two types of patches, source‐sink dynamics are present, and the redistribution of water and nutrients between them improves the availability of resources, thus maintaining ecosystem functioning and stability (Aguiar & Sala, 1999; Reynolds et al., 1999; Schmitz, 2010). Characteristic changes in the spatial organization of vegetation patches are therefore associated with shifts in ecosystem state (Rietkerk et al., 2004; Scheffer et al., 2009). The size distributions of vegetation patches have been observed to follow power law distributions over a wide range of arid regions (Kéfi, Rietkerk, Alados, et al., 2007; Scanlon et al., 2007), indicating self‐organized patterns with many small patches and progressively rare large patches (Pascual et al., 2002). Empirical evidence suggests that the narrowing down of PSDs is associated with environmental harshness (Kéfi et al., 2011; Lin et al., 2010). Deviations of PSDs from pure power laws can be interpreted as indicators of regime shifts or imminent degradation (Kéfi et al., 2014; Scheffer et al., 2009). Therefore, a better understanding of the key drivers for PSD dynamics is vital for the sustainable management of arid ecosystems.

Since high‐resolution remote sensing allows for large‐scale monitoring of vegetation patterns, lognormal distributions have also been demonstrated as an alternative model to power laws for PSDs in arid regions (Meloni et al., 2017; Xu et al., 2015). Based on a global survey of dryland ecosystems, Berdugo, Kéfi, et al. (2019) developed a continuous parameter (power law range, PLR) to quantify the range to which a set of observed patch sizes fits a power law; a power law model is more appropriate if the value of PLR exceeds 0.57, otherwise, a lognormal model is more reliable. Under a drought gradient, the abrupt changes in PLR may reflect the variations of ecosystem functioning, supporting PSDs as indicators of vegetation degradation (Berdugo, Kéfi, et al., 2019; Génin et al., 2018). Moreover, the use of PLR to describe the shape of PSDs allows for a more detailed analysis of biotic and abiotic factors in the spatial organization of vegetation patches (Berdugo, Soliveres, et al., 2017).

A prevailing mechanism for the vegetation patchiness appears to involve a large‐scale resource limitation and short‐range interactions between plants (Rietkerk et al., 2004; Rietkerk & van de Koppel, 2008). Water scarcity is the main constraint to the extension of vegetation patches (Alados et al., 2017; Kéfi, Rietkerk, van Baalen, & Loreau, 2007), while local soil properties such as sand content affecting water redistribution are also associated with the shape of PSDs (Maestre & Escudero, 2009; von Hardenberg et al., 2010). In spatial model analysis, harsh environments tend to be reflected in higher mortality rates for plants during birth–death processes (Kéfi, Rietkerk, Alados, et al., 2007; Sankaran et al., 2019; Xu et al., 2015). Additionally, field investigations have confirmed that environmental conditions contribute to the formation of spatial patterns by influencing biological factors such as total plant cover and plant–plant interactions (Alados et al., 2017; Berdugo, Kéfi, et al., 2019). The elucidation of these links and their effect on PSDs will help unravel the underlying mechanisms in the context of vegetation patchiness being controlled by both the environment and self‐organization (Sheffer et al., 2013).

The outcome of positive and negative plant–plant interactions can lead to different spatial vegetation patterns (Pascual et al., 2002). Facilitation underpins the power law‐like PSDs, whereas competition results in deviations from power laws (Manor & Shnerb, 2008). According to the stress gradient hypothesis, competition prevails in relatively benign environments and shifts to facilitation in stressful environments (Brooker et al., 2008; Maestre et al., 2009). Plant–plant interactions may, however, be modulated more by the traits (e.g., plant height associated with light competition) of the interacting species than by environmental conditions (Soliveres et al., 2014). In shrubland, shrubs with higher canopy heights are able to ameliorate the local environment, thereby creating important mechanistic pathways for facilitative effects (Bråthen & Lortie, 2015). Adapted to the environment, the height of dominant species represents the basic units of vegetation patches at the community‐level (Berdugo, Soliveres, et al., 2017; Moles et al., 2009). Nevertheless, the direct and indirect effects of these traits on PSDs have not been adequately considered.

Here, we undertook a field survey of 80 plots to evaluate the direct and indirect effects of environmental conditions and biological factors on the PSD of the Alxa plateau in Northwest China. A multimodel inference procedure was performed to determine the best predictors of total plant cover, community plant height, plant–plant interactions, and PLR. Using structural equation modeling, we examined the direct and indirect pathways with the best environmental and biological predictors. The specific questions we asked were: (1) how did the environment directly and indirectly drive the spatial organization of vegetation patches in the Alxa plateau, and (2) being an important functional trait, did community plant height directly affect the shape of PSDs, and (3) whether it mediated the environment and affected plant–plant interactions in PSD formation?

## 2 MATERIALS AND METHODS

### 2.1 Study area

**FIGURE 1:**
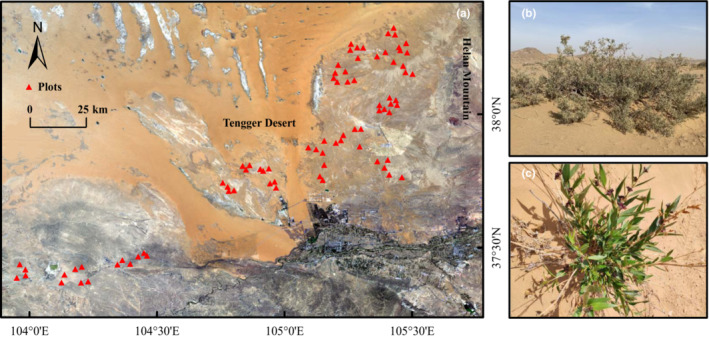
An overview of the study area and the observation plots (a), and representative images of shrub patch (b) and herb patch (c).

This study was conducted in the southeastern part of the Alxa plateau (104°12′–105° 30′ E, 37° 21′–38° 18′ N), bordering the southwestern foot hills of the Helan Mountains and the southeastern margin of the Tengger Desert (Figure 1a). The climate in the area is typical of a continental climate with cold winters and hot summers (Shao et al., 2016). The average annual temperature ranges between 6.0°C and 8.5°C. The mean annual precipitation varies between 45 and 215 mm, of which approximately 70% falls between June and September. Over a range of 3000–4700 mm, the potential evaporation increases from southeast to northwest. The average annual frost‐free period is 156 days (Chen, 2010). The soil types are gray desert soil and gray‐blown desert soil (Ma & Wang, 2020). The vegetation type is a warm temperate desert (Hou, 1983). Communities include monocultures and mixtures, with monocultures consisting primarily of *Reaumuria soongorica (pall.) Maxim*. or *Oxytropis aciphylla Ledeb*., while mixtures consisting of *R. soongorica (pall.) Maxim*. and *Salsola passerine Bunge* or *Ammopiptanthus mongolicus (Maxim.) Cheng f*. and *Zygophyllum xanthoxylum Maxim*.

### 2.2 Field measurement and drone image processing

A total of 80 observational plots (quadrat: 15 m × 15 m) were selected along the gradient of aridity with a variety of taxa and soil types. Plots were selected with flat topography and located more than one kilometer apart. A combination of drone image and field survey was used to investigate the vegetation. A relatively larger area (>225 m^2^) of each plot was scanned by a DJI Phantom® 4 drone with GPS RTK (Da‐Jiang Innovations, DJI, China) in May 2021. The flight route was planned via DJI GS Pro. The flight altitude was 15 m. The lateral and longitudinal overlaps were 80%, meaning that the image acquisition overlapped up to 80% for both *x*‐ and *y*‐axis. The original images with a spatial resolution of 0.4 cm/pix were mosaicked via Agisoft PhotoScan V1.3 in order to produce an orthoimage (Supporting information Appendix D) that has a refined spatial resolution of <0.1 cm/pix (Prošek & Šímová, 2019). We defined a vegetation patch as any shrub and perennial herb (individual and cluster of individuals) that could be identified from the orthoimages and surrounded by bare ground (Figure 1b,c). Vegetation and soil patches were classified in the eCognition 9.0 software by setting different optimal brightness thresholds for orthoimages. We imported the vegetation patches into ArcGIS 10.3, where they were again visually modified and the area of each vegetation patch was calculated to represent the patch size.

By revisiting all the plots with the orthoimages in July 2021, we identified the species composition for each vegetation patch and recorded the number of individuals for each species. In the meantime, the height of 30 mature and healthy individuals for each shrub and perennial herb species forming the patches were measured, and the mean height for each species was calculated (Supporting information Appendix A). To validate the estimation of patch sizes from orthoimages, we measured the longest and shortest diameters of the canopy for each vegetation patch in 10 randomly selected plots and estimated the patch sizes as ellipses. Paired t‐tests were performed to compare the field measured vegetation patch sizes to the software calculations (ArcGIS 10.3) of the 10 plots (Supporting information Appendix B), which showed nonsignificant differences (*p* > .05).

### 2.3 Soil variables and aridity

At the uppermost layer (0–5 cm) in the open areas of each plot, six soil samples were randomly collected. The soil organic carbon (SOC) was determined by the K_2_Cr_2_O_7_–H_2_SO_4_ oxidation method and pH was measured by a pH meter (Sartorius PB‐10) with a 1:2.5 soil to water suspension (Bao, 2000). For particle size analysis, the soil samples were pretreated with H_2_O_2_ (30%, w/w) and by adding sodium hexametaphosphate (NaHMP), sonicating for 30 s (De et al., 2008; Gao et al., 2014). After that, the particle size distribution was determined using a laser granularity instrument (Malvern MS 2000). The proportion of soil particle size >0.05 mm was calculated as the soil sand content. Based on the WorldClim database, we calculated an aridity index (AI = 1 − precipitation/potential evapotranspiration, Berdugo et al., 2020) for each plot.

### 2.4 Patch size distribution

In all the plots, PSDs were fitted to power law distributions of the form (Clauset et al., 2009):

(1) $$p\left(x\right)=\frac{\alpha -1}{x_{\min}}\times {\left(\frac{x}{x_{\min}}\right)}^{-\alpha }$$

where x is the patch sizes, α is the exponent. Since a power law probability density diverges at zero, a minimal threshold $x_{\min}$ needs to be estimated for the tails of PSDs to behave as power laws. The patch sizes below $x_{\min}$ was discarded in fitting, resulting in different sample sizes. The exponent was estimated using maximum likelihood estimation at different $x_{\min}$. As a result, a Kolmogorov–Smirnoff statistic was applied to obtain the optimal fit. The power law range is given as:

(2) $$\begin{array}{c} \mathrm{PLR}=1-\frac{\log_{10}\left(x_{\min}/x_{\text{smallest}}\right)}{\log_{10}\left(x_{\text{largest}}/x_{\text{smallest}}\right)} \end{array}$$

where $x_{\text{smallest}}$ and $x_{\text{largest}}$ is the smallest and largest patch size per plot, respectively. Here, we can see that PLR varies from 0 to 1, representing a relative range for the fraction of remaining part of PSDs that follows a power law (Supporting information Appendix D). A larger value indicates a more representative power law behavior for PSDs (Berdugo, Kéfi, et al., 2019).

### 2.5 Community plant height

The plant height at the community level was characterized by the community‐weighted mean and variance (Enquist et al., 2015), respectively. They are given as:

(3) $$\begin{array}{c} {\mathrm{CWM}}_{j}=\sum_{i}^{n}p_{i,j}h_{i} \end{array}$$

(4) $$\begin{array}{c} {\mathrm{CWV}}_{j}=\sum_{i}^{n}p_{i,j}\left(t_{i}-{\mathrm{CWM}}_{j}\right) \end{array}$$

where *n* is the total number of species sampled in plot *j*, *p*_*i,j*_ is the relative abundance of species *i* in plot *j* calculated by referring to the sum of the abundances for all patch forming species in the plot, *h*_*i*_ is the mean height of species *i*.

### 2.6 Plant–plant interactions

We employed the mark correlation function calculated as the normalized mark pair density to infer plant–plant interactions at short distances (<5 m, Getzin et al., 2008; Illian et al., 2007). The patch sizes were attached to the patch locations as quantitative marks. The test function of mark pair density is $p_{i}\times p_{j}$, where $p_{i}$ and $p_{j}$ are the patch sizes of two points *i* and *j* that are distance *r* apart. The normalization is squared mean patch size per plot and pair density at distance *r*. Thus, the quantitative marked correlation function focuses on the correlation between two patch sizes at a certain distance, while separating it from the effect of patch locations (Law et al., 2009). The null model is independent marking, which assumes that patch sizes are randomly distributed between the locations. We performed 999 simulations of the null model and took the 25 highest and lowest values of the function as envelopes with an error rate of 0.05 (Wiegand et al., 2013). We also used Goodness‐of‐fit to test the probability of type I error (Grabarnik et al., 2011), a departure of empirical observation from the null model was accepted once it reached significant level (*p* < .05).

With flat terrain and small observed plots, we assumed a homogeneous environment within a plot. Therefore, the significant departure was only considered as evidence of plant–plant interactions (Stoyan & Penttinen, 2000; Velázquez et al., 2016). At short distances, if the empirical observations are significantly greater than the simulated envelopes, this is interpreted as plants developing well in size due to facilitation (e.g., microclimate amelioration by above‐ground biomass, Trautz et al., 2017). If the empirical observations are significantly smaller, indicating plants may be limited in sizes due to competition for water or light by neighboring patches (Getzin et al., 2008; Schenk & Jackson, 2002). We used two binary indicators to represent the presence of facilitation and competition (or absence) per plot, respectively. The analysis was carried out in Programita software (Wiegand & Moloney, 2014).

### 2.7 Statistical analysis

Generalized linear models (GLMs) were used to determine the best predictors of community plant height, total cover, plant–plant interactions, and PLR. For community plant height and total cover, the predictors were aridity and soil variables (SOC, pH and sand content); for plant–plant interactions, the predictors increased community plant height and total cover; for PLR, all variables were involved in the prediction. Because plant–plant interactions are binary variables, we used GLMs with a family of binomial distributions. In order to obtain the best models, a model selection procedure was applied based on minimizing the Akaike's Information Criterion corrected for small sample size (AICc, Burnham & Anderson, 2002). The averaged coefficients of predictors were computed by averaging models with ΔAICc (difference from the minimum AICc) less than two units. As none of the two‐way interactions between environmental conditions (climate and soil) and biological factors (community plant height, total cover and plant–plant interactions) significantly affected PLR, we removed all the interactions and only considered the major effects. This procedure was performed by the package MuMIn in R 4.0.2 (Bartoń, 2022).

A structural equation model (SEM) was used to explore the direct and indirect effects of environmental conditions and biological factors on PSDs. We established a priori model (Supporting information Appendix E) based on the previous literatures (Supporting information Appendix F). This model included: (i) a direct effect of environmental conditions, community plant height, and plant–plant interactions on PLR; (ii) a direct effect of environmental conditions on community plant height, total cover, and plant–plant interactions; and (iii) the influence of community plant height and total cover on plant–plant interactions. Based on the best model selection process described above, the potential pathways for the environmental and biological factors to drive PSDs were explored, thereby supporting our a priori model and benefiting the simplification of SEM (Carvajal et al., 2022; García‐Palacios et al., 2018; Wang et al., 2022). We included only the best predictors and removed the hypothesized pathways that were not effective. The SEM was bult by the piecewiseSEM package in R 4.0.2 (Lefcheck, 2016). To assess the goodness‐of‐fit of SEM, Fisher's C statistic was used, with significant values (*p* < .05) indicating that the model cannot fit the data. The standardized total effect of each explanatory variable was also calculated in order to demonstrate the total impact of each variable.

## 3 RESULTS

### 3.1 Optimal predictors for patch size distribution and biological factors

**FIGURE 2:**
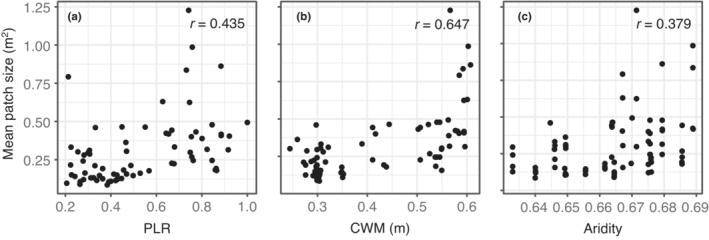
Correlations between the mean patch size and power law range (a), community‐weighted mean height (b), and aridity (c).

The number of patches in the investigated plots varied from 23 to 506. PLR exceeded 0.57 for 35 plots, and it was less than 0.57 for 45 plots (Supporting information Appendix C). The mean patch size was significantly correlated with PLR, community‐weighted mean height and aridity (Figure 2, *p* < .05).

The best predictors for PLR were aridity, community‐weighted mean height and competition between plants (Table 1). Total cover was not affected by aridity, but was positively correlated with SOC. Community‐weighted mean height increased significantly when soil sand content and aridity increased (*p* < .05). Community plant height variance was only positively correlated with soil sand content. As community‐weighted mean height increased, the possibility of competition between plants decreased, whereas the effects of all explanatory variables were insignificant for facilitation between plants (*p* > .05).

**TABLE 1:** Averaged coefficients from the best models for power law range, total cover, community plant height, and plant–plant interactions.

Response variables	Explanatory variables	Estimate	SE	*z*‐value	*p*‐value
PLR	Aridity	**0.27**	**0.09**	**2.96**	**.003**
	CWM	**0.43**	**0.11**	**3.93**	**<.001**
	Competition	**−0.44**	**0.18**	**2.42**	**.016**
	Cover	−0.08	0.07	1.23	.220
	Soil pH	0.09	0.08	1.15	.252
	Facilitation	−0.15	0.13	1.14	.255
Cover	SOC	**0.54**	**0.20**	**2.69**	**.007**
	Sand	0.28	0.22	1.29	.198
	Soil pH	−0.11	0.12	0.92	.355
CWM	Aridity	**0.44**	**0.09**	**4.75**	**<.001**
	Sand	**0.56**	**0.15**	**3.69**	**<.001**
	SOC	0.23	0.15	1.49	.135
CWV	Soil pH	0.18	0.11	1.61	.108
	Sand	**0.58**	**0.16**	**3.57**	**<.001**
	SOC	0.17	0.19	0.90	.367
Facilitation	CWM	−0.56	0.30	1.84	.065
	Soil pH	−0.46	0.32	1.43	.153
	CWV	−0.34	0.28	1.19	.234
	Aridity	−0.43	0.33	1.30	.195
	Cover	0.24	0.26	0.92	.358
	Sand	0.24	0.37	0.66	.511
Competition	CWM	**−3.15**	**1.07**	**2.95**	**.003**
	CWV	−1.11	0.58	1.91	.056
	SOC	−0.66	0.50	1.33	.184
	Aridity	0.80	0.60	1.32	.186
	Cover	−0.46	0.45	1.03	.301
	Soil pH	−0.30	0.38	0.79	.427
	Sand	−0.14	0.92	0.16	.876

Abbreviations: CWM, community‐weighted mean height; CWV, community‐weighted height variance; PLR, power law range; SOC, soil organic carbon.

Bold values indicate *p* < .05 and are considered significant.

### 3.2 Relationship among drivers of patch size distribution

**FIGURE 3:**
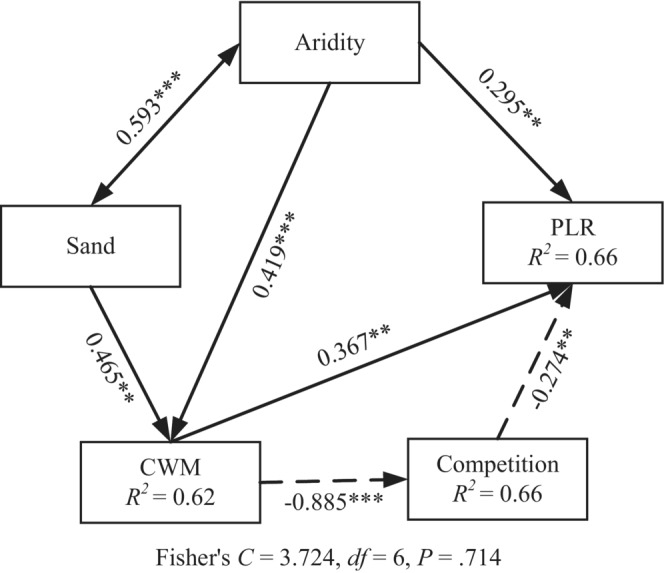
Effects of aridity and biological factors on patch size distributions. One‐headed arrows indicate directional influences, with continuous and dashed lines denote positive and negative effects, respectively. Double‐headed arrow represents the correlation between two variables. Numbers near arrows are standardized path coefficients (*p < .05, **p < .01, ***p < .001). *R* ^2^s of response variables are given in boxes. CWM, community‐weighted mean height. PLR, power law range.

We removed the pathway in our prior model regarding the indirect effect of total cover on PLR since total cover did not affect plant–plant interactions. The simplified SEM supported the direct and indirect effect of environmental and biological factors on PSDs (Fisher's *C* = 3.724; *df* = 6; *p* = .714). Aridity, community‐weighted mean height and competition between plants directly affected PLR (Figure 3). Aridity was positively correlated with soil sand content, and they both directly affected community‐weighted mean height, thereby influencing competition and PLR indirectly. Competition was negatively correlated with community‐weighted mean height, indicating a positive indirect effect of community‐weighted mean height on PLR.

## 4 DISCUSSION

In our study area, two types of PSDs existed at the community level under an aridity gradient. The shape of PSD was directly affected by aridity, community mean height, and competition between plants. Community mean height mediated the effect of aridity on PSDs, while indirectly affecting PSDs by influencing competition.

Spatial vegetation patterns emerge from the interplay between plants and their environment (Alados et al., 2017; Pascual et al., 2002). In arid ecosystems, water is the most limited resource (Aguiar & Sala, 1999; Kéfi, Rietkerk, van Baalen, & Loreau, 2007). Bare soil exhibits properties such as sand content and soil organic carbon that are associated with water infiltration and retention, thereby altering the spatial organization of vegetation patches (Berdugo, Soliveres, et al., 2017; Pugnaire et al., 2004). Soil pH is related to microbial activity, which in turn affects plant–soil feedback (Pugnaire et al., 2019). Our results showed that PLR was influenced indirectly by soil sand content and both directly and indirectly affected by aridity. Meanwhile, aridity was positively correlated with local soil sand content. This indicated that the shape of PSDs in our study area was mainly controlled by climate.

The deviations of PSDs from power laws have been shown to reflect an increase in environmental stress (Kéfi et al., 2011; Kéfi, Rietkerk, Alados, et al., 2007). However, a global survey did not find a significant relationship between aridity and PLR (Berdugo, Soliveres, et al., 2017). According to a recent review of spatial vegetation patterns, the transition from a power law distribution to a regular pattern (e.g., spot patterns) may not indicate ecosystem instability as once believed (Rietkerk et al., 2021). In the present study, aridity positively affected PLR. In the area of relative lower aridity in the Alxa plateau, annual herbs could flourish during the rainy season (June to September). Our survey did not include them in the composition of vegetation patches, which may partially influence the trend of PSD under a climate gradient (Xu et al., 2015).

Importantly, we found that community‐weighted mean height, as a mediator of aridity, had a significant positive effect on PLR. Community‐weighted height variance, however, had no significant effect on PLR. Community functional traits are primarily determined by the local environment (Bruelheide et al., 2018). The mean plant height of the communities in our study area increased with increasing aridity and sand content. This may be a result of the spatial distribution of shrub species. Large shrubs such as *A. mongolicus (Maxim.) Cheng f*. and *C. korshinskii Kom*. are generally found on the margin of the Tengger Desert (Xiao et al., 2019), while dwarf shrubs such as *R. soongorica (pall.) Maxim*. and *S. passerina Bunge* are found in the proluvial fan of the Helan Mountains (Ma & Wang, 2020, 2021). Accordingly, communities located in harsher environments were dominated by large shrubs. Perhaps it is due to the long‐term adaptation of the dominant species to fluctuations in available resources in arid environment (García‐Palacios et al., 2018), and the development of morphological traits that facilitate the acquisition and conservation of resources (Maestre et al., 2021). Shrubs that are large have a stronger ability to ameliorate microhabitats, such as reducing wind erosion, increasing water infiltration, and collecting nutrients under their canopy (Lee, 1991; Wang et al., 2011; Ward et al., 2018).

Our results showed that community‐weighted mean plant height was correlated with mean patch size, indicating that the height of dominant species is representative of the typical characteristics of PSDs (Berdugo, Soliveres, et al., 2017). Clearly, depending on PLR calculation, whether a PSD best fits a power law or a lognormal distribution is related to the maximum and minimum of patch sizes (Berdugo, Kéfi, et al., 2019). Here, we found that PLR was correlated with mean patch size, and mean patch size was also correlated with aridity. It implied that the two types of PSDs coexisted in our study area under the influence of climate, and at the community level they also exhibited distinct variations in community morphological traits.

At short distances, positive or negative correlations between vegetation patch sizes indicate that they depend on each other (Erfanifard et al., 2021; Getzin et al., 2008). The positive correlations indicate facilitation between plants such as reducing wind desiccation by above‐ground biomass (Trautz et al., 2017). The negative ones indicate competition related to water and light sharing by neighbors (Getzin et al., 2008; Schenk & Jackson, 2002). In our study, short‐distance facilitation was not sufficient to form large patches that underpin the creation of power law‐like PSDs. However, competition led to more fragmented patches and PSDs were better fitted to lognormal distributions, which is in accordance with previous model analyses (Manor & Shnerb, 2008; Xu et al., 2015).

High vegetation cover has been shown to increase the frequency of plant–plant facilitation (Meloni et al., 2019; Xu et al., 2015). In our plots, the cover increased significantly with SOC, but was generally less than 30% and did not affect plant–plant interactions. Environmental heterogeneity was not associated with local facilitation between plants, but indirectly affected competition. This indicated that competition became less intense in harsher environments, following the stress gradient hypothesis (Maestre et al., 2009). Interestingly, the probability of competition decreased significantly with increasing plant height. Plant height is associated with competition vigor, primarily for light (Pérez‐Harguindeguy et al., 2013). However, taller shrubs may also provide shelter for other plants by shading effect and reducing wind erosion (Bråthen & Lortie, 2015; Soliveres et al., 2014). In general, facilitation and competition are operating simultaneously, and the net outcome depends on their relative strength (Brooker et al., 2008). Thus, a plausible reason for our results is that the nurse plant effect due to increased plant height may overcome the negative effect of competition for light. Although plant–plant facilitation was not significantly enhanced, the net outcome shifted from negative to neutral as community plant height increased. Furthermore, correlated with other size traits such as root depth and lateral spreading, shrub height may reflect the allometry and partitioning of above‐ and below‐ground biomass (Ma & Wang, 2020, 2021). It has also been demonstrated that competition between shrub species with laterally extended root systems can lead to narrower PSDs (Sheffer et al., 2013; von Hardenberg et al., 2010). However, we only measured community plant height and discussed the effect of functional traits on subsurface competition with some limitations.

Briefly, in addition to the direct effect of community mean height on PLR, it had an indirect effect on PLR by affecting competition between plants. Although point pattern analysis was applied to inferring plant–plant interactions here, previous studies often used the frequency of beneficiary species under nurse plants to identify the relationship between plants (Soliveres et al., 2014; Xu et al., 2015). As a matter of fact, larger shrubs have been found to have stronger nurse plant effects (Peláez et al., 2019; Ward et al., 2018). We, therefore, suggest focusing on functional traits such as plant height and root‐system morphology at the community level in future studies concerning the size distributions of vegetation patches in drylands to better understand the underlying mechanisms of PSD dynamics.

## 5 CONCLUSION

According to our results, the shape of PSDs was primarily affected by aridity, soil sand content, community mean height, and competition between plants in the Alxa plateau, Northwest China. Aridity directly drove PSD formation and was correlated with soil sand content, and indirectly affected competition and PSDs by modulating community plant height. Community plant height acted as a mediator of aridity while affecting competition, both of which were critical for the spatial organization of vegetation patches. Our work suggests that by considering community functional traits as key drivers of PSDs and their connections to local environments and plant–plant interactions, it is promising to deepen the understanding of the underlying mechanisms.

## AUTHOR CONTRIBUTIONS

**Tian‐liang Cheng:** Conceptualization (equal); data curation (equal); formal analysis (equal); investigation (equal); writing – original draft (equal). **Yan‐xia Pan:** Data curation (equal); formal analysis (equal). **Xin‐ping Wang:** Conceptualization (equal); funding acquisition (equal); methodology (equal); writing – original draft (equal); writing – review and editing (equal). **Yan Li:** Funding acquisition (equal); writing – review and editing (equal).

## CONFLICT OF INTEREST STATEMENT

The authors declare that they have no known competing financial interests or personal relationships that could have appeared to influence the work reported in this paper.

## Supporting information


**Appendix S1:** Supporting Information

## Data Availability

The data are available in figshare https://doi.org/10.6084/m9.figshare.20294337.
