# Supplementary material for: Community plant height modulated by aridity promotes spatial vegetation patterns in Alxa plateau in Northwest China
Source: Ecol Evol. 2023 Feb 14;13(2):e9823. doi: 10.1002/ece3.9823 (PMC9929261; doi:10.1002/ece3.9823)
Supplement: Supplementary file 1 — Appendix S1: Supporting Information [file ECE3-13-e9823-s001.docx]

**APPENDIX A**

Mean height of shrub and herb species forming patches.

| Species | Mean height (Standard error) / m |
| --- | --- |
| *Reaumuria soongorica (pall.) Maxim.* | 0.299 (0.004) |
| *Salsola passerina Bunge* | 0.307 (0.006) |
| *Ammopiptanthus mongolicus* *(Maxim.) Cheng f.* | 0.619 (0.015) |
| *Nitraria tangutorum Bobrov* | 0.415 (0.018) |
| *Zygophyllum xanthoxylon* *Maxim.* | 0.568 (0.011) |
| *Oxytropis aciphylla* *Ledeb.* | 0.223 (0.011) |
| *Artemisia ordosica Kraschen.* | 0.502 (0.038) |
| *Kalidium gracile Fenzl* | 0.300 (0.021) |
| *Sympegma regelii Bunge* | 0.266 (0.010) |
| *Caragana korshinskii Kom.* | 0.686 (0.027) |
| *Amygdalus mongolica Maxim.* | 0.567 (0.050) |
| *Potaninia mongolica* *Maxim.* | 0.255 (0.009) |
| *Ceratoides latens (J. F. Gmel.) Reveal et Holmgren* | 0.561 (0.028) |
| *Atraphaxis bracteate A. Los.* | 0.101 (0.019) |
| *Cynanchum mongolicum (Maxim.) Kom.* | 0.289 (0.009) |
| *Achnatherum splendens (Trin.) Nevski* | 0.625 (0.027) |

**APPENDIX B**

Results of paired t-tests between vegetation patch sizes estimated by images and field measurements of randomly selected 10 plots. Plot numbers are in accordance with Appendix C.

| Plot | *df* | *t*-value | *p*-value |
| --- | --- | --- | --- |
| 3 | 49 | 1.4731 | 0.1471 |
| 4 | 126 | 0.9348 | 0.3517 |
| 7 | 111 | 0.5783 | 0.5642 |
| 10 | 77 | 0.9529 | 0.3436 |
| 11 | 117 | 0.7188 | 0.4737 |
| 22 | 195 | 1.1455 | 0.2534 |
| 32 | 72 | 1.8281 | 0.0717 |
| 46 | 41 | 1.8446 | 0.0723 |
| 57 | 86 | 0.7777 | 0.4389 |
| 64 | 163 | -1.6618 | 0.0985 |

**APPENDIX C**

Supplementary data for patch sizes in each plot. Number of patches, mean size and the parameters in Equation 2 are provided.

| Plot | n | Mean  / m^2^ | Xsmallest  / m^2^ | Xlargest  / m^2^ | Xmin  / m^2^ | PLR | Plot | n | Mean  / m^2^ | Xsmallest  / m^2^ | Xlargest  / m^2^ | Xmin  / m^2^ | PLR | Plot | n | Mean  / m^2^ | Xsmallest  / m^2^ | Xlargest  / m^2^ | Xmin  / m^2^ | PLR |
| --- | --- | --- | --- | --- | --- | --- | --- | --- | --- | --- | --- | --- | --- | --- | --- | --- | --- | --- | --- | --- |
| 1 | 259 | 0.109 | 0.009 | 1.425 | 0.185 | 0.4 | 28 | 27 | 0.552 | 0.013 | 3.674 | 0.018 | 0.946 | 54 | 182 | 0.318 | 0.005 | 14.702 | 0.021 | 0.823 |
| 2 | 438 | 0.132 | 0.007 | 1.258 | 0.228 | 0.324 | 29 | 307 | 0.156 | 0.009 | 2.67 | 0.185 | 0.469 | 55 | 122 | 0.241 | 0.011 | 1.022 | 0.29 | 0.278 |
| 3 | 50 | 0.478 | 0.005 | 9.553 | 0.016 | 0.845 | 30 | 103 | 0.163 | 0.018 | 1.201 | 0.361 | 0.286 | 56 | 398 | 0.156 | 0.006 | 1.058 | 0.129 | 0.413 |
| 4 | 127 | 0.423 | 0.012 | 2.829 | 0.381 | 0.368 | 31 | 121 | 0.333 | 0.007 | 6.455 | 0.065 | 0.68 | 57 | 87 | 0.179 | 0.007 | 4.249 | 0.016 | 0.865 |
| 5 | 247 | 0.192 | 0.012 | 1.829 | 0.294 | 0.364 | 32 | 73 | 0.63 | 0.006 | 5.142 | 0.072 | 0.628 | 58 | 181 | 0.096 | 0.005 | 0.577 | 0.212 | 0.206 |
| 6 | 147 | 0.284 | 0.008 | 1.982 | 0.392 | 0.291 | 33 | 51 | 0.836 | 0.02 | 8.339 | 0.102 | 0.732 | 59 | 96 | 0.193 | 0.004 | 5.254 | 0.01 | 0.863 |
| 7 | 112 | 0.363 | 0.006 | 3.56 | 0.188 | 0.466 | 34 | 44 | 0.315 | 0.006 | 3.45 | 0.011 | 0.916 | 60 | 52 | 0.311 | 0.001 | 1.748 | 0.191 | 0.306 |
| 8 | 78 | 0.298 | 0.005 | 7.786 | 0.03 | 0.749 | 35 | 162 | 0.301 | 0.009 | 4.848 | 0.973 | 0.255 | 61 | 166 | 0.114 | 0.005 | 0.558 | 0.077 | 0.421 |
| 9 | 237 | 0.131 | 0.007 | 1.528 | 0.29 | 0.312 | 36 | 96 | 0.305 | 0.004 | 3.04 | 0.141 | 0.469 | 62 | 23 | 0.862 | 0.014 | 17.35 | 0.032 | 0.884 |
| 10 | 78 | 0.272 | 0.004 | 6.92 | 0.013 | 0.847 | 37 | 74 | 0.464 | 0.011 | 6.56 | 0.369 | 0.449 | 63 | 130 | 0.4 | 0.007 | 32.52 | 0.058 | 0.754 |
| 11 | 118 | 0.152 | 0.01 | 5.394 | 0.013 | 0.956 | 38 | 274 | 0.115 | 0.005 | 0.968 | 0.167 | 0.338 | 64 | 164 | 0.177 | 0.007 | 5.801 | 0.129 | 0.568 |
| 12 | 60 | 0.418 | 0.005 | 4.73 | 0.054 | 0.654 | 39 | 143 | 0.183 | 0.009 | 1.063 | 0.121 | 0.456 | 65 | 262 | 0.105 | 0.005 | 0.95 | 0.125 | 0.391 |
| 13 | 170 | 0.151 | 0.008 | 0.827 | 0.291 | 0.227 | 40 | 48 | 0.493 | 0.012 | 6.482 | 0.012 | 1 | 66 | 506 | 0.085 | 0.006 | 0.941 | 0.133 | 0.384 |
| 14 | 89 | 0.424 | 0.017 | 2.006 | 0.088 | 0.658 | 41 | 241 | 0.106 | 0.006 | 0.883 | 0.235 | 0.265 | 67 | 177 | 0.245 | 0.005 | 8.942 | 0.028 | 0.762 |
| 15 | 57 | 0.463 | 0.011 | 6.695 | 0.193 | 0.551 | 42 | 207 | 0.212 | 0.007 | 1.333 | 0.106 | 0.488 | 68 | 101 | 0.46 | 0.024 | 2.198 | 0.489 | 0.332 |
| 16 | 45 | 0.986 | 0.034 | 5.086 | 0.114 | 0.758 | 43 | 106 | 0.287 | 0.006 | 2.243 | 0.363 | 0.307 | 69 | 112 | 0.388 | 0.007 | 5.987 | 0.027 | 0.801 |
| 17 | 146 | 0.161 | 0.009 | 1.203 | 0.092 | 0.529 | 44 | 188 | 0.223 | 0.011 | 1.999 | 0.059 | 0.678 | 70 | 97 | 0.404 | 0.008 | 3.103 | 0.013 | 0.921 |
| 18 | 53 | 0.407 | 0.007 | 3.261 | 0.014 | 0.885 | 45 | 201 | 0.28 | 0.004 | 2.853 | 0.453 | 0.282 | 71 | 71 | 0.167 | 0.005 | 1.251 | 0.012 | 0.832 |
| 19 | 50 | 0.625 | 0.013 | 12.16 | 0.073 | 0.746 | 46 | 42 | 1.227 | 0.012 | 21.416 | 0.085 | 0.742 | 72 | 142 | 0.121 | 0.008 | 1.117 | 0.136 | 0.428 |
| 20 | 62 | 0.432 | 0.018 | 8.492 | 0.072 | 0.774 | 47 | 80 | 0.443 | 0.013 | 4.387 | 0.089 | 0.668 | 73 | 73 | 0.273 | 0.008 | 2.497 | 0.016 | 0.873 |
| 21 | 205 | 0.215 | 0.01 | 1.274 | 0.435 | 0.223 | 48 | 182 | 0.245 | 0.006 | 7.04 | 0.019 | 0.845 | 74 | 69 | 0.423 | 0.01 | 9.482 | 0.117 | 0.643 |
| 22 | 196 | 0.149 | 0.007 | 1.07 | 0.127 | 0.427 | 49 | 162 | 0.146 | 0.008 | 1.036 | 0.09 | 0.498 | 75 | 455 | 0.12 | 0.005 | 0.95 | 0.222 | 0.28 |
| 23 | 178 | 0.147 | 0.005 | 1.492 | 0.207 | 0.35 | 50 | 183 | 0.089 | 0.007 | 0.517 | 0.164 | 0.264 | 76 | 109 | 0.226 | 0.01 | 6.423 | 0.085 | 0.668 |
| 24 | 73 | 0.792 | 0.005 | 5.495 | 1.226 | 0.213 | 51 | 164 | 0.141 | 0.008 | 0.855 | 0.272 | 0.242 | 77 | 50 | 0.387 | 0.01 | 3.524 | 0.025 | 0.835 |
| 25 | 183 | 0.125 | 0.005 | 0.839 | 0.128 | 0.364 | 52 | 183 | 0.332 | 0.013 | 3.533 | 1.006 | 0.225 | 78 | 60 | 0.183 | 0.012 | 1.818 | 0.024 | 0.859 |
| 26 | 223 | 0.211 | 0.008 | 1.677 | 0.289 | 0.33 | 52 | 183 | 0.295 | 0.013 | 3.533 | 1.006 | 0.225 | 79 | 103 | 0.268 | 0.009 | 6.911 | 0.046 | 0.756 |
| 27 | 157 | 0.127 | 0.014 | 0.68 | 0.12 | 0.447 | 53 | 115 | 0.552 | 0.013 | 2.021 | 0.449 | 0.3 | 80 | 41 | 0.416 | 0.006 | 6.565 | 0.014 | 0.885 |

**APPENDIX D**

Examples for the orthoimages of plots (a, b), power law fit (c, d) and mark correlation function (e, f). Images for plant communities dominated by *R. soongorica (pall.) Maxim.* (a, plot 13 in Appendix C) and *A. mongolicus (Maxim.) Cheng f.* (b, plot 32 in Appendix C). Open circles denote the inverse cumulative frequency of patch sizes (c, d). Dashed lines denote estimated $x_{min}$. Red lines show the fitted power law with scaling parameter being 3.564 and 1.582, respectively. Green lines with double arrows denote power law range. Filled circles and dashed lines denote observed mark correlation function (e, f). Grey ribbons denote envelops. Black colored circles denote nonsignificant deviations from the null model, whereas red colored circles denote significant deviations tested by Goodness-of-fit.


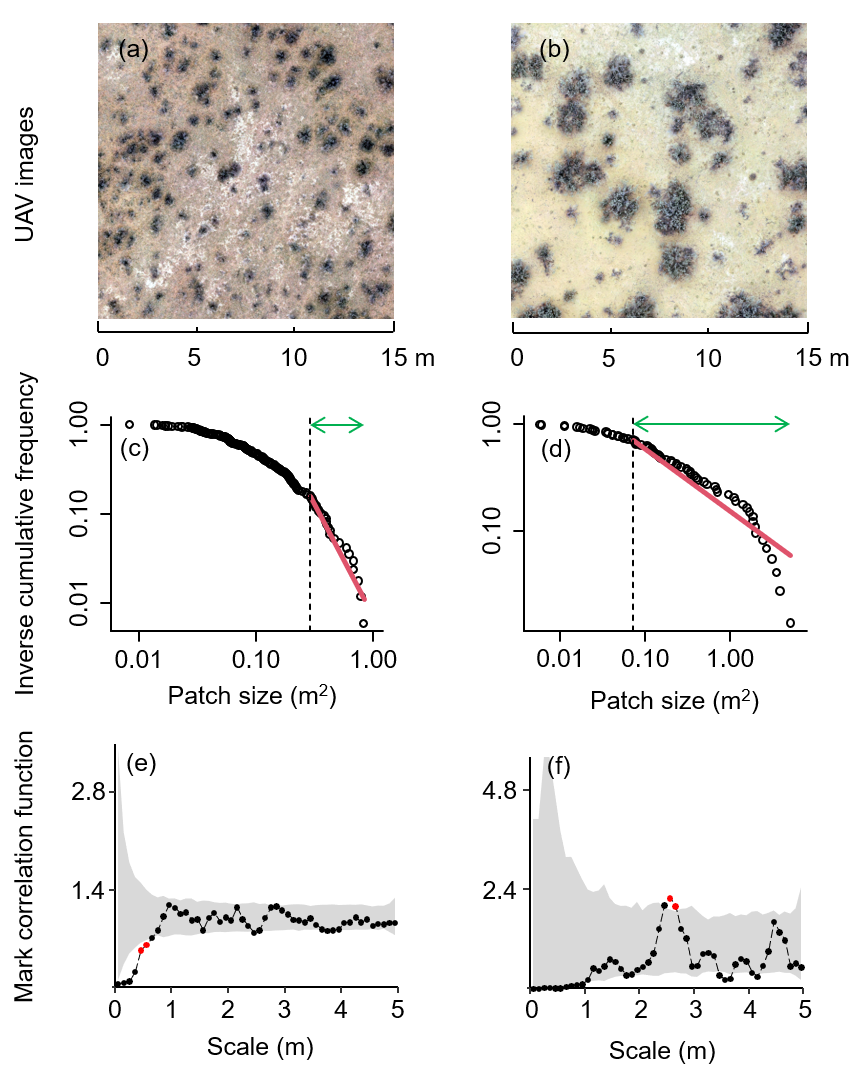


**APPENDIX E**

A priori structural equation model assessing the direct and indirect effects of environment (aridity and/or soil), total plant cover, community plant height (community weighted mean and/or variance), plant-plant interactions (competition and/or facilitation) on the power law range of patch size distributions. Rectangles represent explanatory and response variables. Arrows with numbered circles indicate hypothesized pathways.

**APPENDIX F**

Rational of hypothesized pathways among driving factors and the power law range of patch size distributions in a prior model in Appendix E. References have been included in main text.

| **#** | **Associations** | **Rationale** | **Ref.** |
| --- | --- | --- | --- |
| 1 | Environment → Cover | Water shortage and soil degradation limit the growth of plants. | Kéfi et al. (2007b); Maestre and Escudero (2009) |
| 2 | Environment → Patch size distribution | Resources limitation (water and soil nutrients) cause a long-range competition for vegetation and result in patchiness. | Rietkerk and van de Koppel (2008); Kéfi et al. (2011) |
| 3 | Environment → Plant-plant interactions | Facilitation is more frequent in harsh environments (Stress gradient hypothesis). | Brooker et al. (2008); Maestre et al. (2009) |
| 4 | Environment → Height | ​In response to their habitat, species develop morphological traits associated with resource acquisition and conservation. | Bruelheide et al. (2018); García-Palacios et al. (2018) |
| 5 | Height → Patch size distribution | The plant height of dominate species in a community represents the basic unit of patch sizes. | Berdugo et al. (2017b) |
| 6 | Cover → Plant-plant interactions | An increase in total plant cover can increase the probability of plants getting in touch with each other. | Xu et al. (2015); Meloni et al. (2019) |
| 7 | Height →Plant-plant interactions | Plant height is associated with competition for light. Higher shrubs may provide shade and protection from wind erosion for other plants. | Soliveres et al. (2014); Bråthen and Lortie (2015) |
| 8 | Plant-plant interactions → Patch size distribution | Local interactions between plants could lead to either regular or irregular vegetation patches. | Scanlon et al. (2007); Kéfi et al. (2011) |
